# Scoping Recreational Disturbance of Shorebirds to Inform the Agenda for Research and Management in Tropical Asia

**DOI:** 10.21315/tlsr2020.31.2.4

**Published:** 2020-08-06

**Authors:** Sumudu Marasinghe, Greg D. Simpson, David Newsome, Priyan Perera

**Affiliations:** 1 Department of Forestry and Environmental Sciences, University of Sri Jayewardenepura, Gangodawila, Nugegoda 10250, Sri Lanka; 2 Harry Butler Institute Murdoch University: Centre for Sustainable Aquatic Ecosystems, Murdoch University, Perth, WA 6150, Australia; 3 Sukau Ecotourism Research Center (SERC), BEST Society, Lot 1, Pusat Perindustrian Kolombong Jaya, Jalan Kolombong, 88450 Kota Kinabalu, Sabah, Malaysia; 4 College of Science, Health, Engineering and Education: Environmental and Conservation Sciences, Murdoch University, Perth, WA 6150, Australia

**Keywords:** Ecotourism, Recreational Disturbance, Shorebirds, Sustainable Development Goals, South Asia, Southeast Asia, Tropical Asia

## Abstract

In addition to scoping the impacts of the four most reported sources of recreational disturbance on shorebirds, this study also advances the concept of Tropical Asia (TA) to collectively describe tourist destinations in the ecologically and geopolitically diverse part of the planet that incorporates the tourism megaregion of South and Southeast Asia. At a time of growing global concern about the rapid decline of shorebird populations, many governments in TA are embracing and capitalising on the exponential growth in demand for coastal recreation and tourism across the region. This political response is partly driven by efforts to deliver economic development, aligned to the United Nations Sustainable Development Goals, in order to secure the livelihoods of people living in less developed coastal areas. However, the rapid increase in visitor numbers and the development of infrastructure to support the booming demand for coastal tourism destinations in TA are further exacerbating the pressures on shorebird populations across the region. Despite these growing pressures and the wealth of research reporting on shorebird populations across the Asian flyways, this scoping study identified surprisingly little research that reports on the recreational disturbance (RD) of shorebirds in TA. While undertaken to inform future research, this study also provides a synthesis of management strategies reported in the global literature into a set of management recommendations for coastal destinations in TA.

**Highlights**

- Identifies a lack of research into the recreational disturbance of shorebirds in Tropical Asia and begins to address that gap via findings and management options published in the global literature.
- The four most reported sources for recreational disturbance of shorebirds were pedestrians moving through coastal ecosystems, exercising of pet dogs in coastal habitat, driving motor vehicles in coastal environments, and all forms of inshore recreational boating.
- Impacts on the foraging of shorebirds and reduced breeding and/or reproductive success were the most commonly reported negative outcomes of disturbance from human recreational activities.

## INTRODUCTION

At a time when shorebird populations are in steep decline globally, many Asian governments are embracing an exponential growth in tourism demand to deliver economic development aligned to the United Nations (UN) Sustainable Development Goals (UNSDG n.d.) in order to secure the livelihoods of communities living in coastal areas (Hitchcock *et al*. 2018; Holden 2016; Larson 2015; Lilleyman *et al*. 2018; Ong & Smith 2014; Sachs 2012; UN World Tourism Organization (UNWTO) 2019; Ziegler *et al*. 2018). Many authors report that marine focused recreation and tourism has the potential to be a major market segment for the expansion and promotion of authentic ecotourism based on the rich natural resources of South and Southeast Asia (e.g. Australian Institute of Marine Science 2018; Chon 2013; Newsome 2013; Perera & Vlosky 2013; Senevirathna & Perera 2014; UNWTO 2019). For that reason, countries in South and Southeast Asia have developed as some of the most popular and important ecotourism destinations on the planet (Chon 2013; Hitchcock *et al*. 2010; Newsome *et al*. 2013; Newsome *et al*. 2019). Singapore, Malaysia and Thailand are high demand tourism destinations, of which nature-based tourism (NBT) is a significant component (Newsome & Simpson 2020; Steven *et al*. 2020). Tourism demand for NBT destinations in Indonesia and the Philippines is also growing rapidly (Gaia Discovery 2017; White & Rosales 2003). The nations of Brunei, Cambodia, Myanmar, Sri Lanka and Vietnam are also expanding their tourism offerings to attract developments based on cultural and ecotourism products (Gaia Discovery 2017; Lew 2001). Hereafter, we collectively refer to the regions of South Asia and Southeast Asia as the tourism megaregion of Tropical Asia (TA – see also Methods). According to the UNWTO (2018), tourist arrivals at Asian destinations in the Indo-Pacific region increased by an average 6% for the year 2017. Sandy beaches, coral reefs, islands expressing natural scenic beauty, and rich cultural heritage continue to attract tourists and recreationists to coastal destinations throughout TA (Hitchcock *et al*. 2018; Kunsook & Dumrongrojwatthana 2017; Lück 2007; Newsome *et al*. 2019).

TA also has significant potential for wildlife tourism, especially birdwatching, to supplement existing attractions at coastal destinations (Ismail & Rahman 2016; Li *et al*. 2013; Ma *et al*. 2013). Hundreds of endemic and migratory bird species can be observed, because of the diversity of habits and large number of stopover/staging sites located in the region (e.g. Azman *et al*. 2011; Mansor & Sah 2012; Marasinghe *et al*. 2015, 2018; Perera *et al*. 2017; Rosely *et al*. 2007; Zakaria & Rajpar 2010). As reported in Ma *et al*. (2013), commercialised birdwatching has grown rapidly over the past two decades to become a prominent ecotourism market segment in mainland China alone. However, increasing demand for tourism in TA has created substantial changes and negative impacts with respect to the natural values of coastal environments (Chon 2013; Hitchcock *et al*. 2018; Holden 2016; Onn *et al*. 2009). With rising tourism across the region, attention needs to be given to the protection of the natural environment, and especially with respect to its actual and potential NBT values. Without increased protection of environmental values in TA, ecotourism assets may be lost before their sustainable development potential can be realised (Azman *et al*. 2011; Ismail & Rahman 2016; Newsome 2013; Newsome *et al*. 2019; Perera *et al*. 2017).

Globally, the plethora of beach-based ecotourism and recreational activities that occur in coastal zones are increasingly considered to be major anthropogenic sources of disturbance to shorebirds (Gill 2007; Mayo *et al*. 2015; McFadden *et al*. 2017; Steven *et al*. 2011). Tourism and recreational impacts specific to birds have been previously reported by Buckley (2004), Newsome *et al*. (2005), Sekercioglu (2002) and Steven *et al*. (2011). Those studies report the significant sources of bird disturbance to comprise the alteration or destruction of habitat, alteration of natural behaviours, and/or increased rates of predation and birds being injured and killed. Moreover, recreational disturbance (RD) is viewed as a major threat to shorebird populations (Bregnballe *et al*. 2009; Drewitt 2007; Gill *et al*. 2001; Meager *et al*. 2012; Oldland *et al*. 2009; Schou & Bregnballe 2007; Stillman *et al*. 2007; Trulio & White 2017; Webber *et al*. 2013). In addition to the direct impacts of the rising demand for ecotourism and recreation in coastal zones, the increased installation of facilities and infrastructure to service that demand is exacerbating the impact on shorebird populations (Clark 2018; Sharma & Rao 2018; Yasué & Dearden 2006). The sources and impacts of RD and management recommendations reported in this study are, however, focussed on the activities of tourists or visitors that are associated with and that emanate from such facilities and infrastructure, as well as those people who freely and independently access coastal environments for recreation.

Shorebirds are especially vulnerable to human RD because of their size, behaviours, and physical beauty which tend to attract birders (Carney & Sydeman 1999; Weller 1999). They are disturbed by the recreational activities in coastal ecosystems and are forced to leave the area temporarily or permanently depending on the severity of the disturbance (Geering *et al*. 2007). As such, the impacts from modification or destruction of habitat and alteration of natural behaviours can have significant negative effects at the individual, population, and community level (Azman *et al*. 2011; Bregnballe *et al*. 2009; Fernández-Juricic & Tellería 2000; Gill *et al*. 2001; Mallord *et al*. 2007; Mansor & Sah 2012; Meager *et al*. 2012; Schou & Bregnballe 2007; Webber *et al*. 2013). Cayford (1993) defined the disturbance of shorebirds, specifically waders, arising from recreational activities to be any relatively discrete event in time that disrupts ecosystems, communities or populations, where disruption refers to a change in behaviour, physiology, numbers or survival.

Aware of the regional challenges to shorebirds summarised above, because of our experience researching avifauna and NBT in TA (e.g. Alwis *et al*. 2016; Marasinghe *et al*. 2015, 2018; Newsome 2013; Newsome *et al*. 2019; Newsome & Simpson 2020; Perera & Vlosky 2013; Perera *et al*. 2015, 2017), we undertook this review to inform our future research agenda in this space. Given the wealth of research reporting on shorebird populations across the Asian flyways (e.g. Galbraith *et al*. 2014; Hansen *et al*. 2016; Heim *et al*. 2018; Pearce-Higgins *et al*. 2017; Si *et al*. 2018), we were surprised to discover the lack of reported RD research from TA. Given that lack of research, in this scoping study we summarise the global literature to assist the prioritisation of shorebird focused RD research in TA. To that end, this study explores foot traffic, exercising pet dogs, motorised vehicles, and recreational boating as the four most reported sources of RD on shorebirds in the coastal zone. Further, this study also reports on management actions recommended in the global literature to enhance shorebird protection in coastal environments in TA.

## METHODS

### Rapid Scoping Review

The methods of documentary research that utilise the techniques of systematic literature review is a dynamic and evolving field of inquiry that is now employed across many disciplines (Moher *et al*. 2009; Pickering & Byrne 2014; PRISMA 2015; Simpson & Parker 2018a; Temple University 2019). As mentioned above, we undertook this review to inform our future research regarding the nexus between coastal ecotourism and recreation and the RD of shorebirds in TA. Therefore, we undertook a rapid scoping review with the broad aims of characterising the focus of such research, identifying the primary sources and impacts of RD for shorebirds, and to discover where and what RD research was reported from TA.

Scoping reviews are employed to determine the form and volume of literature that is available for a topic of interest, provide a preliminary synthesis of that literature, and to identify gaps in the existing literature/research for that topic (PRISMA 2015; Temple University 2019). We decided to perform a scoping review, as this technique is particularly suited to situations where the existing literature has not been comprehensively reviewed in the context of the proposed research or where the literature is large, complex, and/or heterogeneous in nature (Temple University 2019). We faced both scenarios with respect to our attempt to characterise the coastal RD literature with respect to TA. The synthesis from a scoping review can be reported in a tabulated and/or in a descriptive form (Temple University 2019), as we report in this study. Scoping reviews are often undertaken to determine if a full systematic review of the literature is warranted and to inform the research questions for such a review (PRISMA 2015; Temple University 2019). With standard systemic reviews typically taking 12+ months to complete, rapid reviews are a documented adaption of the systematic review techniques undertaken to generate a timely synthesis of the evidence when short deadlines apply, or resources are limited (Temple University 2019).

For the scoping review reported in this study, the online databases Web of Science, Google Scholar, JSTOR, Emerald Insight, and Science Direct were searched to identify articles that reported on the effects of RD on shorebirds in coastal ecosystems. The search was performed using the terms ‘coastal birds’, ‘shore birds’, ‘shorebirds’, or ‘waders’ in combination with the terms ‘impact of ecotourism’, ‘nature-based tourism’, ‘recreational disturbance’ and ‘behavioural responses’. The temporal range for the search was restricted to research published between the 1 January 2000 and 31 December 2018, because we were interested in contemporary research, especially with respect to research from TA.

**Figure 1:**
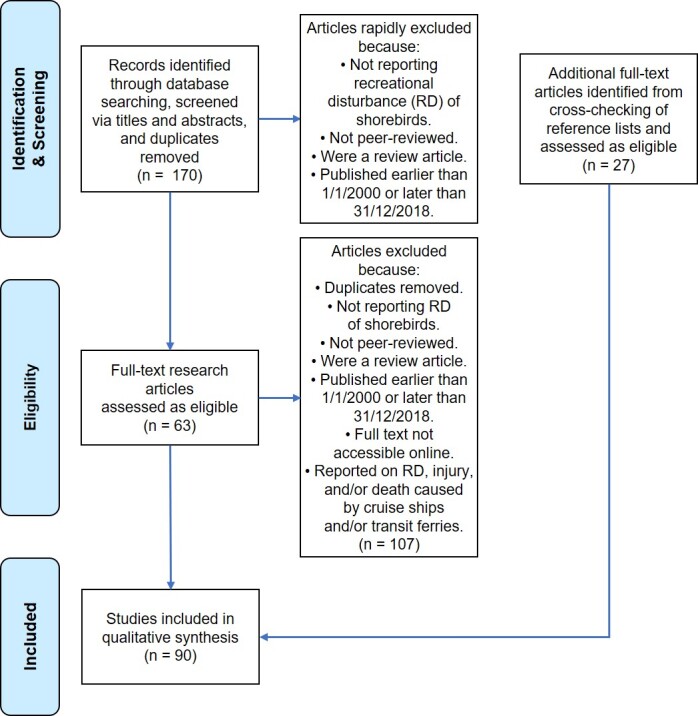
Preferred Reporting Items for Systematic Reviews (PRISMA) expression for the rapid scoping review.

Because of the volume of publications reporting on avifauna research that were identified by the searches, the titles and abstracts of identified publications were screened for phrases that related to RD research and coastal environments to ensure that the most relevant articles were considered for the review. This screening was performed concurrent with the searches that identified the articles (Fig. 1). Publications that were selected after the identification/screening stage were considered for inclusion in the scoping review based on the following inclusion/exclusion criteria. Thus, to be included in this scoping review, publications had to be:

1. Peer-reviewed articles reporting the findings of original research (i.e. review articles and grey literature were excluded);
2. Published in English language journals; and
3. Accessible online as a full-text article.

Further, we also took the decision to exclude articles that reported acute negative impacts caused by large vessels, such as cruise ships and transit ferries (including high-speed ferries), because we believed that RD was a secondary factor in those human-bird interactions.

Subsequently, the reference lists of included articles were scanned for additional research articles reporting studies related to the topic and meet the inclusion/exclusion criteria reported above and in Fig. 1 and one further article by Ramli and Norazlimi (2017) was identified during the peer-review process was also included in the review.

The relevant findings reported in the included articles were then extracted and analysed. The extracted data was analysed based on: was the research conducted in TA or elsewhere, reported source(s) of RD, reported impact(s) of RD, and any recommendations for minimising RD. The synthesis of that analysis is reported in the Findings and Discussion section.

### Defining TA

As mentioned above, the purpose of this study was to inform future RD research and the management of RD impacts on shorebirds in South and Southeast Asia. Many diverse terrestrial and maritime nations straddle this boundary between Greater Asia and Oceania, across the arc linking the northern Indian Ocean to the western Pacific Ocean via the Indonesian Throughway (Gaither & Rocha 2013; UN Statistics Division 2019). Numerous biogeographic, climatic, floristic, oceanographic, zoogeographic, and geo-political regions have been proposed to cluster and describe this region (e.g. Brummitt 2001; Cox 2001; Morgan 1984; UN Statistics Division 2019).

**Figure 2:**
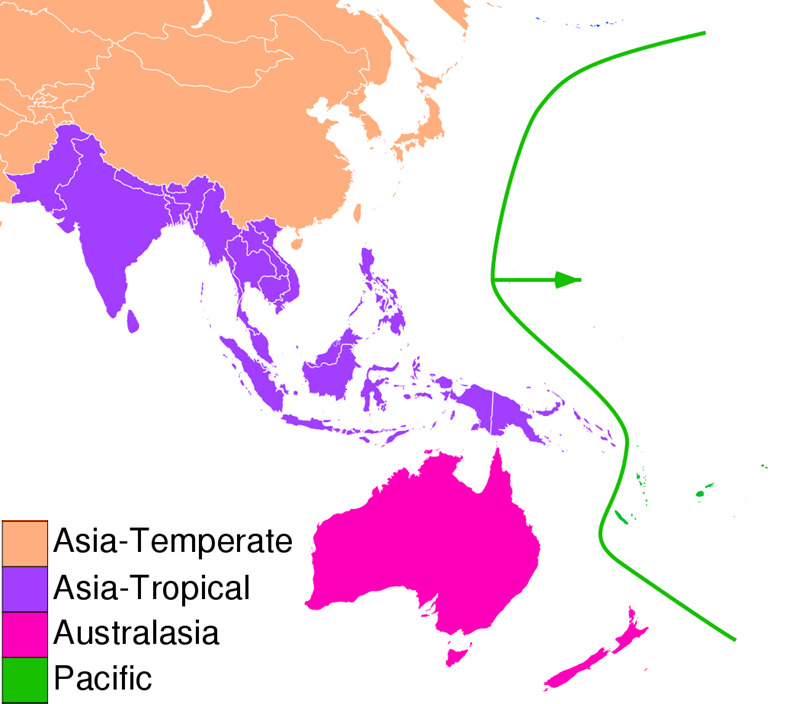
The TA biogeographical region (Adapted from Coxhead 2015).

Given the lack of a precise definition, we advance Tropical Asia as the collective proper noun to describe this emerging tourism megaregion that is being actively targeted both by tourists and by tourism researchers. We posit that the flora-oriented province of ‘Asia – Tropical’ proposed by Brummitt (2001, pp. 13–15), as originally adopted by the International Working Group on Taxonomic Databases (TDWG), best gathers the marine and terrestrial tourist destinations of this region, especially in the context of NBT research (Fig. 2).

### Defining the Coastal Zone

At a finer scale, coastal zones are areas where both fresh and saline water and the land surface interact, creating distinct and diverse environments (Burke *et al*. 2001). While a long established and commonly used phrase, definitions and understandings of what constitutes the coastal zone remain a contested concept (Blackburn *et al*. 2019; Western Australian Planning Commission [WAPC] 2003). Davis and FitzGerald (2009) defined the coast to be any segment of the earth that is influenced by marine conditions such as salinity, tides, winds and biota. Features of coastal zones are soft-shores, rocky shores, cliffs, narrow or wide coastal shelves, hilly or flat coastal plains, and various types of wetlands including freshwater lakes, saltmarshes, estuaries and deltas (Donaldson *et al*. 1995 (sic for 1994) cited in WAPC 2003; Schwartz 2005). Based on ecological and physical inter-connections, coastal zones may extend further inland to encompass watersheds and rivers that drain into coastal waters (Beatley *et al*. 2002; Carter 2013; Simpson & Newsome 2017). Coastal habitats are identified as valuable and important ecosystems, due to both goods and ecosystem services provided to humans as well as to the environment (Carter 2013; Simpson & Newsome 2017).

## FINDINGS AND DISCUSSION

### RD Research from TA

Of the 90 articles included in our scoping review (Fig. 1), just three of those (3%) report RD research from TA (Choi *et al*. 2015; Ramili & Norazlimi 2017; Yasué & Dearden 2006). The apparent scarcity of RD research in the Asian flyways identified by the scoping review that informs this study is surprising. Completion of a full systemic review would identify all the literature from TA and more fully inform the setting of the RD research agenda for the region. However, the summary of the global RD research reported below provides the platform and impetus for this much needed research in TA. Further, this study can also inform practitioners as to best practice management to minimise the impacts of RD on shorebirds until more research specific to TA becomes available.

### Impact and Magnitude of RD

As previously alluded to and reported in detail below, the four sources of RD for shorebirds most commonly reported in the included global literature are the presence of humans, exercising pet dogs, the operation of motor vehicles, and recreational boating. Most of the included studies report on the impact of RD on the foraging behaviour of shorebirds, with those studies reporting that foraging of shorebirds is negatively affected by the human activities (Albores-Barajas & Soldatini 2011; Burger *et al*. 2004; Lafferty 2001a; Martin *et al*. 2015; Trulio & Sokale 2008). According to a study conducted at the Pacific Rim National Park on Vancouver Island, British Columbia by Yasué (2006), shorebirds were found to respond more to human disturbance when the ecological cost of foraging was lower, and that displaced shorebirds returned quickly when they were feeding in habitats with high prey availability and during the late afternoon. However, other studies have shown that birds that are forced to fly from place to place, because of continuous human disturbance, risk energy losses that pose a risk to bird survival (e.g. Hvenegaard & Barbieri 2010). An example is, the endangered migratory Black-faced Spoonbill (*Platalea minor*) that winters in East Asia (BirdLife International 2001; Chen *et al*. 2010). At a wintering ground on Jeju Island, Republic of Korea, the Spoonbill is under threat of local extinction, because increased levels of tourism are intensifying the incidence of birds being flushed. The resultant abrupt and unexpected escape flight activity is postulated to negatively affect the finely tuned winter energetic balance of the birds (Choi *et al*. 2015). In addition to reduced energy budgets impacting on the health and survival of adult shorebirds, the loss of foraging time and feeding opportunities can also lead to adult birds not being capable of meeting the energy demands of successfully raising young at summer breeding grounds (e.g. Leseberg *et al*. 2000).

The combination of reduced breeding and/or reproductive success were the next most reported outcomes for the impact of human recreational activities on shorebirds. Birds in coastal environments and island settings are susceptible to disrupted courtship, displacement from nests, and chicks being exposed to predators (documented in Newsome *et al*. 2005, 2013). Similarly, using examples from three diverse species, RD was found to negatively impact the breeding success of Yellow-eyed penguins (*Megadyptes antipodes*), American oystercatchers (*Haematopus palliates*), and Kentish plovers (*Charadrius alexandrinus*) (Ellenberg *et al*. 2007; Martin *et al*. 2015; Sabine III *et al*. 2008). Reported causes for reduced reproductive success, as a consequence of RD impacting chick survival, include nest abandonment, reduced foraging of adults during brood rearing, reduction of food delivered to chicks, separation of one or more chicks from rest of the brood, and forcing the broods into less suitable habitats (Albores-Barajas *et al*. 2009; Albores-Barajas & Soldatini 2011; McClung *et al*. 2004; Ruhlen *et al*. 2003; Sabine III *et al*. 2008).

### Disturbance from Foot Traffic

Habitat selection and habitat use by shorebirds can be altered by the presence of humans (Burger & Niles 2013; Lafferty 2001a; Madsen *et al*. 2009; Ramili & Norazlimi 2017), and many studies report birds being readily disturbed when approached by people (e.g. Lafferty 2001a; Koch & Paton 2014; Reyes-Arriagada *et al*. 2013; Sabine III *et al*. 2008; Trulio & White 2017). As is often the case in ecological studies however, not all bird species are equally affected by RD and the response of shorebirds can vary by location, situation, and/or species (Choi *et al*. 2015; Yasué & Dearden 2006).

Shorebirds can exhibit variable responses to crowds of people in the coastal zone (Ramili & Norazlimi 2017). Hvenegaard and Barbieri (2010) and Martin *et al*. (2015) reported on a negative relationship between the number of tourists and the abundance of shorebirds. Conversely, Stigner *et al*. (2016) found that crowding did not affect shorebird abundance. Similarly, an earlier study by Gill *et al*. (2001) reported that while shorebirds exhibited avoidance behaviour in the presence of humans, there was no reduction in the number of birds in the study area. Forcing a bird to fly is the most significant negative impact of shorebirds being disturbed into avoidance behaviour, as flight requires a greater expenditure of energy during the escape (Blumstein 2003; Fernández-Juricic *et al*. 2001).

The Flight Initiation Distance (FID), which is the distance at which birds take flight as an escape behavioural response, is one measure that can be used to assess disturbance in the presence or according to the actions of humans (Barter *et al*. 2008; McLeod *et al*. 2013; Stankowich & Blumstein 2005). McLeod *et al*. (2013) examined the FID of waterbirds in regard to vehicles (see later), bicycles, and walkers highlighting the complexity of waterbird response in regard to species affected and the type of activity. It has been found that some species are able to distinguish between different activities, for example, vehicle versus human pedestrian presence. McLeod *et al*. (2013), however, cautioned that the extent, location and frequency of the different stimuli needed to be taken into consideration when implementing management actions. Glover *et al*. (2011) investigated 36 regularly occurring shorebird species in Australia and reported that FID is significantly influenced by factors such as starting distance of human approach, previous exposure to humans, flock size, and type of the stimulus (e.g. walker, jogger, walker with dog). Moreover, higher approach speeds may cause greater disturbances to birds (Mayo *et al*. 2015), thus birds are more disturbed by joggers than walkers (Glover *et al*. 2011). A further complication in determining the impact of avoidance behaviours relates to the sensitisation or habituation of shorebirds to the presence of humans. Research by Lafferty (2001b) from the United States of America that considered 57 species at a Californian beach reported evidence of shorebirds becoming more sensitised by disturbance, with the average distance at which shorebirds reacted to human presence increasing with the amount and frequency of disturbance within a particular day. In contrast, Lord *et al*. (2001) observed that the avoidance behaviour of New Zealand Dotterel decreased with repeated exposure to human visitations. Similarly, Ikuta and Blumstein (2003) and Martínez-Abraín *et al*. (2008) report that the avoidance behaviour of several species of shorebirds decreases after repeated exposures, despite increasing numbers of visitors.

### Disturbance from Exercising Pet Dogs

There has been a significant increase in the ownership of pet dogs over the past two decades, including in the countries of TA, and this has led to the incidence of owners seeking out ‘dog friendly’ destinations (Boost *et al*. 2017; Christian *et al*. 2017; Galay *et al*. 2018; Parker & Simpson 2018a; Ramili & Norazlimi 2017; Simpson & Parker 2018b). The review of Christian *et al*. (2017) reported that dog owners find aesthetically pleasing reserves and nature spaces to be their preferred locations to exercise their dogs. Further, the presence of ‘natural wildlife’ increased the motivation of owners to exercise their dog(s) at those locations, because they perceived the presence of wildlife ‘to be supportive of a dog walking’ philosophy (Christian *et al*. 2017). As a result, people accompanied by their dogs have become an increasingly significant subset of the RD of shorebirds from human foot traffic. There are now a number of papers reporting that dogs pose a major threat to shorebirds. Shorebirds are impacted by owners exercising their pet dogs in the coastal zone due to dogs chasing the birds, disruption of foraging, disruption of nesting and incubation, and the predation of eggs and chicks (Burger *et al*. 2004; Lafferty 2001a; Leseberg *et al*. 2000; Lord *et al*. 2001). Further, Glover *et al*. (2011) have documented that shorebirds perceive a walker with a dog as a greater threat than a walker alone, hence birds responded to dogs at greater distances and with higher intensities than to a human walking alone. Accordingly, the abundance of shorebirds is reported to decline as the number of dogs being exercised increases and the presence of owners and dogs was found to have twice the disturbance impact of people walking without dogs (Stigner *et al*. 2016).

### Disturbance from Motor Vehicles

Motor vehicles (with two or more wheels) traversing coastal zones are a significant source of RD for birds on beaches in countries such as Australia, Canada England, Norway, Sri Lanka, Thailand, and the United States of America (Schlacher *et al*. 2013; Shashikala & Perera 2018). Schlacher *et al*. (2013) reported that such vehicular disturbance leads to frequent, energy sapping, and time-consuming escape behaviours in coastal zone birds. Vehicles traversing beaches also have a negative impact on the foraging time of shorebirds, as the time they spend on vigilance and responding to disturbance stimuli is increased by the presence of vehicles, which increases with proximity (Stolen 2003; Sih *et al*. 2011). The presence of vehicles also negatively affects the foraging rates of shorebirds and can displace birds from favourable feeding and roosting sites (Meager *et al*. 2012; Ramili & Norazlimi 2017; Stolen 2003;). Regarding the specifics of vehicle activity, the proximity and changes in the movement of the vehicles can intensify the magnitude of RD. Great Egrets (*Ardea alba*) and Snowy Egrets (*Egretta thula*), for example, decrease foraging when vehicles stop adjacent to feeding birds or when vehicles were driven slowly to observe the birds (Stolen 2003). The same study demonstrated that close proximity of the disturbing vehicle also influences the probability of flushing shorebirds. Vehicular traffic may also negatively affect reproductive success during the chick-rearing phase, due to stressed chicks leaving the nest, adults abandoning nests and chicks, and/or nests and chicks being crushed (McGowan & Simons 2006). Bird collision with vehicles is another reported consequence of increased recreational vehicle traffic in the coastal zone. In an example of a negative feedback loop, adult shorebirds, chicks, and nests and eggs are at an increased risk of being run-over by vehicles traversing beaches through the maladaptation of birds resting by crouching in vehicle ruts made by the repeated passage of vehicles (Schlacher *et al*. 2013).

### Disturbance from Recreational Boating

Recreational boating encompasses the use of a diverse range of watercraft including sailing dinghies and yachts; motorised boats of all sizes; human powered craft, such as canoes, kayaks, and stand-up paddleboards (or SUPs); windsurfers and kiteboards; powered personal watercraft (or PWCs - also known as jet-skis); and water-skiers and people being towed behind powerboats on floatation devices. As previously reported in the Methods, the scoping review reported in this study excluded research into commercial operations that use boats, ferries, and larger vessels to transport tourists around and between coastal destinations.

It has been observed that the general behaviour of shorebirds in coastal environments is adversely affected by recreational boating. Reduced foraging and feeding is a commonly reported effect of RD from boats (e.g. Bellefleur *et al*. 2009; Merkel *et al*. 2009; Velando & Munilla 2011). As for foot traffic and motorised vehicles, the disturbance of shorebirds by recreational boating directly impacts their energy budget through the loss of feeding opportunities. Displacement of birds from optimal foraging areas is another consequence of water-based recreational activities (Velando & Munilla 2011). Some species attempt to compensate for lost feeding opportunities by feeding during high tides. This behaviour further compromises their energy budget since the cost of feeding is higher when the water is deeper, and the width of the feeding zone reduced (Merkel *et al*. 2009).

Chick survival rates are also negatively impacted by recreational boating activities. Speckman *et al*. (2004) reported that fish holding adult Marbled Murrelets (*Brachyramphus marmoratus*) swallowed the fish intended for their chicks because of disturbance from skiff sailing boats. It was also postulated that if adults had to make a lot of repeat foraging trips, due to this type of disturbance, there may be a substantial energy cost to the adult and even bigger cost to chicks. Agness *et al*. (2008) showed that foraging Kittlitz’s Murrelets (*Brachyramphus brevirostris*) most commonly respond to vessels by diving regardless to the size, distance, and the speed of the approaching boat. Agness *et al*. (2008) also reported that near-shore density of Kittlitz’s Murrelets had declined as a result of boat disturbance.

The literature indicates that all forms of recreational boating are a disturbance threat for birds in the coastal zone (e.g. Agness *et al*. 2008; Beale & Monaghan 2004; Burger & Niles 2013; Chan & Dening 2007; Frid & Dill 2002; Jenkins 2002; Madsen *et al*. 2009; Merkel *et al*. 2009; Peters & Otis 2005; Speckman *et al*. 2004; Suski & Cooke 2007). The magnitude of disturbance that shorebirds experience from recreational boating is dependent on the type of boat, speed of the boat; frequency and level of the noise produced; distance and direction of boat approach; and the number of boats present (Burger & Niles 2013; Le Corre *et al*. 2013; Ronconi & St. Clair 2002; Velando & Munila 2011). While it may seem obvious that PWCs would contribute to the disturbance of shorebirds (Chan & Dening 2007; Rodgers & Schwikert 2002), the presence of watercraft such as canoes and kayaks, which may seem relatively innocuous in the marine environment, have also been reported to disturb shorebirds (Chatwin *et al*. 2013).

As reported by Rodgers and Schwikert (2002) the type of recreational boat influences the RD of shorebirds, with both large outboard-powered boats and PCWs eliciting greater flushing responses from larger shorebird species than for other types of boat. Rodgers and Schwikert (2002) further reported that the greater flushing response resulted from the noise generated by larger outboard-powered boats and the large vertical and horizontal spray commonly produced by PWC. With the capacity to operate at high speed in shallow water the RD created by both these two types of watercraft can have major negative effects for shorebirds foraging and loafing in shallow waters.

Not surprisingly, the number of boats present at a site has also been found to affect the behaviour of shorebirds. Velando and Munilla (2011) investigated boat disturbance on European Shags (*Phalacrocorax aristotelis*) and found that an increase in the number of boats at a marine reserve was associated with increased spatial aggregation of the Shags, exclusion of Shags from the best feeding areas, and concentration of the birds into areas with little traffic. Velando and Munilla (2011) also reported that foraging activity of Shags decreased by ten times when the number of boats anchored at the study location exceeded 50, because the birds ceased foraging and become alert whenever a moving boat entered the location.

Similarly, Merkel *et al*. (2009) observed that when heavily disturbed by recreational boating, the feeding activity of Common Eider ducks (*Somateria mollissima*) decreased by 60% and the daily locomotion of the birds tripled. Bellefleur *et al*. (2009) investigated boat disturbance on Marbled Murrelets (*Brachyramphus marmoratus*) and reported fewer birds foraging in areas with high boat traffic. Moreover, behavioural changes due to high frequencies of passing boats can impose energetic constraints on birds (Bright *et al*. 2003). In support, Mayo *et al*. (2015) found a positive correlation between flight response and the number of boats operating in the area.

The distance from the passing boat also influences the RD effect for shorebirds. Merkel *et al*. (2009) reported that distance to the boat creating the RD was a significant explanatory variable for the disruption to feeding activity of Common Eider ducks. Similarly, Bellefleur *et al*. (2009) reported that the proportion of shorebirds reacting to disturbance from boats increased when the distance to source of the RD decreased. Moreover, direction of the approaching boats also affects the response of shorebirds. Burger *et al*. (2010) showed that Black skimmers (*Rynchops niger*) allowed the boats moving tangentially to colony to approach closer than boats approaching directly.

The speed of boat approach is a major determinant for the magnitude of the impact of RD on shorebirds. The proportion of birds being flushed and flushing distance are both greater with increased speed of the approaching boat (Bellefleur *et al*. 2009). In contrast to the impacts from recreational boats with motors, the research of Chatwin *et al*. (2013), on several species of shorebirds around Vancouver Island, Canada, reported that a kayak could approach significantly closer than the motorboats without causing RD.

### Management Recommendations

The level of protection afforded to the flora and fauna of a coastal destination is dependent on the degree of planning and management applied to human use and activities in the natural environment (Makino *et al*. 2013; Newsome & Moore 2015). Key strategies aimed at protecting shorebirds include appropriate coastal zone policy and environmental protection legislation. Implicit in this is the designation of suitable protected areas that can act as safe feeding, resting and breeding sites for birds (e.g. Holden 2016; Newsome *et al*. 2005; Newsome *et al*. 2013; Orams 1999). Tourism planning can be applied that caters for recreational activities but avoids conflicts. Applying the Spectrum of Marine Recreation Opportunities (SMARO) described by Orams and Lück (2014) can assist in identifying compatible and incompatible uses in coastal settings.

Given the popularity of coastal destinations for ecotourism and recreational activities in TA, several strategies are therefore required to minimise the negative effects of RD on shorebirds in the region (Table 1). Furthermore, given the species and population specific responses to RD exhibited by shorebirds, it is essential that consideration is given to the responses of individual shorebird species to differing sources and magnitudes of disturbance when determining management strategies (Glover *et al*. 2011; Martin *et al*. 2015; Stigner *et al*. 2016).

Zoning of recreational activities on beaches and other shorebird habitat in coastal zones is one such strategy that conservation and land managers in TA can use to reduce the effect on RD on shorebirds (Newsome *et al*. 2005, 2013; Orsini *et al*. 2006; Schlacher *et al*. 2013; Stigner *et al*. 2016). Zoning can be used to limit or constrain recreational activities to specific areas and/or to control or restrict visitor access from important shorebird habitat, such as breeding colonies (Burger & Gochfeld 2007; Newsome *et al*. 2013). Alternatively, a higher level of protection can be provided by the creation of sanctuary zones that totally restrict access (e.g. areas where boats, dogs, motor vehicles, and/or humans are prohibited); or to allow low impact ecotourism and recreation (e.g. birdwatching from constructed hides, limited number of tightly regulated eco cruises conducted in electric powered small boats) in well managed areas that are providing refugia for shorebirds (Sabine III *et al*. 2008; Stolen 2003). A further consideration are the suggestions of Choi *et al*. (2015) and Ismail and Rahman (2016) that maintaining buffer zones around key habitats is essential to ensure the effect of RD in shorebirds is minimised.

Several strategies based on a combination of administrative controls and/or the installation of physical infrastructure (Tables 1 and 2) have proven successful in restricting or controlling the access of pedestrians and motor vehicles to critical coastal habitats and/or sensitive sites that shorebirds utilise during key life cycles stages (e.g. mating, nesting, chick hatching and rearing). To varying extents, the appropriate and consistent implementation of these strategies, singularly or in combination, have been shown to be effective and successful measures in minimising RD and enhancing shorebird conservation.

In addition to the management strategies indicated in Tables 1 and 2, it is recommended to restrict or ban dogs from ecologically important coastal habitat that shorebirds utilise for nesting, roosting, breeding and foraging (Lafferty 2001a, 2001b; Lord *et al*. 2001; Stigner *et al*. 2016). Such initiatives can, however, be controversial among dog owners (Ham *et al*. 2008). In addition to being a contentious issue from the perspective of the protection and conservation of shorebirds, the presence and behaviour of dogs being exercised at coastal locations can impact the motivation of non-dog walkers to visit these natural areas to enjoy the experience and gain the health benefits of connecting with nature (Christian *et al*. 2017; Ham *et al*. 2008; Parker & Simpson 2018a, 2018b). Further, dissatisfaction with a natural area tourism experience, such as the dissatisfaction experienced by non-dog walkers who are exposed to dogs being exercised by other people, reduces the levels of recommendation and re-visitation by ecotourists and negatively impacts on destination image, which is especially so for people interested in seeing shorebirds as part of their visit to coastal zones in TA (Agius *et al*. 2018; Newsome & Simpson 2020; Parker & Simpson 2018a; Perera & Vlosky 2017).

It has been suggested that, buffer zones with reduced/controlled boating activity should be introduced around sensitive bird areas (Bellefleur *et al*. 2009; Rodgers & Schwikerts 2002). According to Agness *et al*. (2008), large and fast-moving vessels cause a greater disturbance to birds. Application of speed limits for boating activities and managing the number of boats at a location (Bellefleur *et al*. 2009; Velando & Munilla 2011) can be used as strategies to mitigate negative effects on shorebird populations.

The inappropriate and/or ill-considered perceptions, attitudes, and actions of people are one of the main impediments to wildlife conservation and the appropriate human appreciation of nature (e.g. Newsome *et al*. 2005; Simpson *et al*. 2016; Patroni *et al*. 2019). Awareness raising and educating visitors about coastal destinations in TA regarding the importance of conserving shorebirds, the effects of human-centred recreational behaviours, and how to reduce the adverse effects RD is essential to prevent localised and more widespread species extinction (Antos *et al*. 2007, Ismail & Rahman 2016; Schlacher *et al*. 2013; Stolen 2003). Notwithstanding the aspects considered above, the research of Patroni and others (2018, *et al*. 2018a, *et al*. 2018b, *et al*. 2019) reports that visitors participating in ecotourism experiences at coastal destinations are both concerned for the welfare of wildlife and want to be educated about the wildlife of the area. Implementation of location specific awareness and education programmes (see Table 1) is essential in TA, because of rapid development, tourism and increasing visitor pressure in coastal zones. The outcomes of this scoping review demonstrate that there is lack of research regarding visitor perceptions and attitudes in relation to the effects of RD on shorebirds in TA. Well-planned on-site interpretation (see Table 1) can play a vital role in this regard (Newsome 2013; Newsome *et al*. 2013; Perera & Vlosky 2017). The above-mentioned on-site tourism management strategies, which are currently employed at many sites around the world, can be adopted by planning authorities and conservation managers in TA to enhance policy development, coastal zone planning and conservation outcomes for shorebirds, which are critical ecotourism assets for the region.

Implementation of the management recommendations synthesised by this study can directly contribute to UNSDG 8 – Decent Work and Economic Growth; UNSDG 11 – Sustainable Cities and Communities; UNSDG 12 – Responsible Consumption and Production; UNSDG 14 – Life below Water; and UNSDG 15 – Life on Land. Furthermore, by enhancing community and economic development through the implementation of ecologically sustainable development and ecotourism in the coastal zones of TA. Moreover, this article can indirectly contribute to UNSDG 1 – No Poverty; UNSDG 9 – Industry, Innovation and Infrastructure; and UNSDG 17 – Partnerships for the Goals (Sachs 2012; UNSDG n.d.; UNWTO, 2019).

## CONCLUSION

Many governments in TA are embracing an exponential growth in tourism that is, in part, being driven by the expansion and promotion of marine focused ecotourism, based on the rich natural resources of the region, to deliver economic development aligned to the UNSDG that can secure the livelihoods of communities living in coastal areas. This development comes at a time of growing global concern about the rapid decline of shorebird populations and it is likely that the booming demand for coastal recreation and tourism in TA will exacerbate the pressures on shorebird populations across the region. However, despite the wealth of research reporting on declining bird populations across the Asian flyways, the scoping review that informed this study suggests a scarcity of recreational RD research from TA. In addition to providing a summary of the global literature that can inform and prioritise this much needed research in TA, this study also provides recommendations that can inform best practice management to reduce the impacts of RD on shorebird populations in TA.

The four most reported sources of RD that impact shorebird populations are foot traffic, exercising pet dogs, operating motor vehicles, and recreational boating. Focused conservation efforts should, therefore, be introduced to protect shorebird communities in TA from the impacts of the global phenomenon of an exponential increase in coastal recreation and tourism. While it is not possible to eliminate all impacts on shorebirds due to coastal recreation and tourism (and other development/human exploitation related factors), the impacts of RD should be minimised by the designation and protection of important bird habitats in coastal zones and the application of a suite of management strategies. Those management approaches include: maintaining buffer zones around key habitats; zoning recreation activities in specific areas separated from sites such as breeding colonies that are critical for shorebird conservation; using physical barriers as a visitor management tool, temporary closure of beaches or some areas of coastal habitats during the periods when shore birds are most vulnerable; and using observation hides to ensure that tourists are invisible to shorebirds.

Increasing RD research in TA will provide the evidence-based scientific approach needed to inform the management of recreational and tourism activities in coastal habitats and the economic and ecological cost of the impacts of the RD of shorebirds must be considered as part of a coordinated agenda for coastal conservation policy and planning across the region.

## Figures and Tables

**Table 1:** Techniques for managing RD to shore birds.

Management Approach a	Implementation Strategies a
Policy and legislation	Protected area designation Adequate protected area management Managing tourism development
Regulatory techniques	Limit visitor numbers, Controls on numbers and access Prohibit certain activities, Regulate scale and frequency of interaction Close areas to use/zoning Separate activities/zoning
Physical techniques	Site hardening Installations of boardwalks, viewing platforms and/or bird hides Facility design and placement Rehabilitation of mangroves and coastal wetlands
Economic techniques	Differential fees (discounted boat ramp fee for off peak use) Damage bond (financial incentive for good practices) Fines (penalty for inappropriate behaviour) Rewards (prizes for sustainable tourism initiatives and practices)
Educational techniques	Printed material Signs Visitor centre/s Guided walks/talks Activities Personal contact with tourists/visitors Managing tourist behaviour (education and supervision)

a Derived from Orams (1999), Newsome et al. (2005) and Newsome et al. (2013).

**Table 2:** Strategies for restricting and/or controlling the access of pedestrians and motor vehicles to minimise the effects of RD on shorebird at coastal recreation and tourism destinations in TA.

Access Management Strategy	Source(s)
Boardwalks and/or designated pathways to control low impact access.	Burger & Gochfeld (2007); Newsome *et al*. (2013)
Physical barriers to permanently restrict access.	Burger & Gochfeld (2007); Ikuta & Blumstein (2003); Hvenegaard & Barbieri (2010); Mayo *et al*. (2015)
Temporary closure of beaches or some areas of coastal habitats.	Burger & Niles (2013); Schlacher *et al*. (2013)
Strategic distribution of beach access points.	Lafferty (2001a)
Building bird-viewing platforms.	Hvenegaard & Barbieri (2010)
Installing observation hideouts, screenings and shelters to ensure that visitors are invisible to shorebirds.	Burger *et al*. (2004); Bregnballe *et al*. (2009); Holm & Laursen (2009)
